# Cyclopentenylcytosine (CPE-C): In Vitro and In Vivo Evaluation as an Antiviral against Adenoviral Ocular Infections

**DOI:** 10.3390/molecules28135078

**Published:** 2023-06-29

**Authors:** Eric G. Romanowski, Kathleen A. Yates, Y. Jerold Gordon

**Affiliations:** The Charles T. Campbell Ophthalmic Microbiology Laboratory, UPMC Vision Institute, Department of Ophthalmology, School of Medicine, University of Pittsburgh, Pittsburgh, PA 15213, USA

**Keywords:** antiviral, adenovirus, cyclopentenylcytosine, eye, conjunctivitis, in vitro, animal model

## Abstract

Adenoviruses are the major cause of ocular viral infections worldwide. Currently, there is no approved antiviral treatment for these eye infections. Cyclopentenylcytosine (CPE-C) is an antiviral that has demonstrated activity against more than 20 viruses. The goals of the current study were to determine the in vitro and in vivo antiviral activity of CPE-C as well as its ocular toxicity. Antiviral activity was evaluated in vitro using standard plaque reduction assays to determine the 50% effective concentrations (EC_50_s) and in vivo in the Ad5/NZW rabbit ocular replication model. Ocular toxicity was determined in uninfected rabbit eyes following topical ocular application. The in vitro EC50s for CPE-C ranged from 0.03 to 0.059 μg/mL for nine adenovirus types that commonly infect the eye. Ocular toxicity testing determined CPE-C to be non-irritating or practically non-irritating by Draize scoring. In vivo, 3% CPE-C topically administered 4X or 2X daily for 7 days to adenovirus-infected eyes demonstrated effective antiviral activity compared with the negative control and comparable antiviral activity to the positive control, 0.5% cidofovir, topically administered twice daily for 7 days. We conclude CPE-C was relatively non-toxic to rabbit eyes and demonstrated potent anti-adenoviral activity in vitro and in vivo.

## 1. Introduction

Human adenovirus (HAdV) is the major cause of sporadic and epidemic ocular viral infections worldwide [1]. These ocular infections, in the forms of epidemic keratoconjunctivitis (EKC), follicular conjunctivitis, and pharyngeal conjunctival fever, produce significant patient morbidity and have led to the closure of clinics and hospitals in Japan [2]. Patients lose valuable time from school and work during acute infections [1]. The most severe form of adenoviral ocular infections is EKC as this involves both the conjunctiva and cornea causing some patients to develop chronic vision problems (impaired vision, glare) due to persistent immune infiltrates in the cornea that can last from months to years [1]. Pseudomembranous conjunctivitis can also be associated with EKC [1]. EKC is mostly caused by the Species D adenoviruses HAdV8, HAdV19, and HAdV37 [1].

Follicular conjunctivitis is the mildest form of adenoviral ocular infection [1]. Follicular conjunctivitis involves only the conjunctiva producing hyperemia of the bulbar conjunctiva and a follicular response on the tarsal conjunctiva [1]. These infections can be caused by almost any adenovirus type, but are predominantly caused by adenovirus types HAdV3, HAdV7a, (Species B), and HAdV4 (Species E) [1]. Pharyngeal conjunctival fever is a disease seen mostly in children. It is characterized by a sore throat, enlargement of the preauricular lymph nodes, and associated conjunctivitis. It is most commonly caused by adenovirus type HAdV3 and several other types to a lesser extent [1]. All of these acute infections are self-limiting, but there is significant morbidity associated with them. Therefore, a treatment for these acute viral infections is considered an unmet medical need in ophthalmology. 

At present, there is no approved antiviral treatment anywhere in the world that has proven to be effective against these eye infections. While various compounds have demonstrated antiviral efficacy against adenovirus in the laboratory [3,4], none have undergone successful development to reach the clinic as a therapeutic option. Among the most promising, cidofovir (S-HPMPC), a nucleoside analog antiviral that inhibits adenovirus DNA polymerase, was successfully tested in preclinical studies [5,6,7,8] and in Phase 1 and 2 clinical trials in the USA for the treatment of adenoviral ocular infections [9]. However, toxicity (epiphora due to secondary lacrimal canalicular blockade) led to the discontinuation of the development of topical cidofovir in the USA [10]. Furthermore, two small clinical trials testing cidofovir in combination with cyclosporine A in patients with adenoviral conjunctivitis produced inconclusive results on the antiviral efficacy of topical cidofovir [11,12]. Cidofovir is US FDA- and EMA-approved for the intravenous treatment of cytomegalovirus retinitis in AIDS patients.

Several studies ranging from preclinical to clinical trials have been reported over the last 15 years with other promising anti-adenoviral agents including 2′,3′-dideoxycytidine (ddC, zalcitabine, a pyrimidine nucleoside analog) [13,14,15], topical Iv-IgG (human immunoglobulin G) [16], doxovir (CTC-96, a cobalt-chelating agent) [17], N-chlorotaurine (NCT, a weak halogen-based oxidant) [18,19], povidone-iodine [20,21], FST-100 (a combination of 0.4% povidone-iodine and 0.1% dexamethasone [22,23], auricloscene (NVC-422, N,N-dichloro-2,2-dimethyltaurine, a novel analog of NCT) [24,25], ganciclovir (a synthetic analog of 2-deoxyguanosine) [26,27], filociclovir [28] (a novel methylene-cyclopropane nucleoside analog), astodrimer sodium (a novel dendrimer) [29], and ranpirnase (a ribonuclease) [30]. However, none of these agents has yet received US FDA or EMA approval and the world still awaits an effective drug to meet an unmet medical need that affects millions of patients worldwide annually.

**Figure 1:**
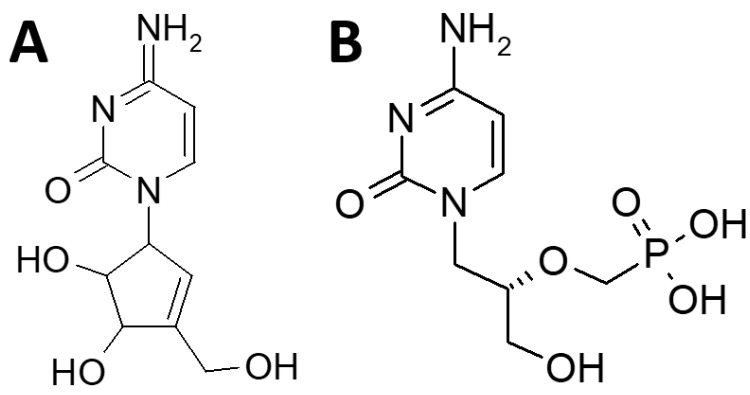
This figure presents the chemical structures of (**A**) CPE-C and (**B**) cidofovir. Both are nucleoside analogs of cytosine.

Cyclopentenylcytosine (CPE-C) is a carbocyclic nucleoside and the synthetic analog of the naturally occurring fermentation product of neplanocin A (Figure 1A) [31]. It is a nucleoside analog of cytosine, similar to cidofovir (Figure 1B), and was originally synthesized as an anticancer drug. Although both CPE-C and cidofovir are cytosine analogs (Figure 1), they possess different structures and mechanisms of action. CPE-C (Figure 1A) is a carbocyclic nucleoside analog of cytosine in which the sugar part is replaced by a cycloalkyl ring [32]. The carbocyclic ring makes it resistant to degradation by nucleoside phosphorylases that cleave the usual N-glycosidic bond [32]. The general mechanism of action of CPE-C is mediated through the inhibition of the enzyme CTP synthetase, the enzyme that converts UTP to CTP. This interaction results in a depletion of the CTP supply necessary for viral DNA replication [32].

In contrast, cidofovir (Figure 1B) is an acyclic nucleoside phosphonate antiviral. During DNA replication, nucleosides are phosphorylated by host cell kinases into their active triphosphate form, which is then taken up by polymerases and incorporated into the growing DNA chain [33]. The same process is required for nucleoside analogs. A limitation of using nucleoside analogs as antivirals is the specificity of the kinases involved in the phosphorylation steps [33]. The addition of a phosphonate moiety in cidofovir increases the stability of the phosphate bond which delivers a monophosphate mimic into the cell, thereby overcoming the first phosphorylation step [33]. After a second phosphorylation step, the diphosphate form of cidofovir thereby acts as a competitive inhibitor and an alternative substrate during the DNA polymerase reaction [34].

Two patient deaths from refractory hypotension during clinical trials ended the development of CPE-C at that time for the treatment of solid tumors [35]. CPE-C was also reported more than 35 years ago to have broad-spectrum antiviral activity against more than 20 RNA and DNA viruses, including HSV, CMV, VZV, and yellow fever virus [31,32]. More recently, interest in CPE-C was renewed as a potential inhibitor of West Nile Virus [36] and De Clercq has stated that CTP inhibitors represent a class of potential antivirals that have not been fully assessed; “especially their in vivo potential remains to be explored” [37].

The goals of the current study were to determine the antiviral activity and ocular toxicity of CPE-C for potential development as an anti-adenoviral ocular therapy. Specifically, CPE-C was evaluated for efficacy in vitro against a broad panel of common ocular adenovirus types and in the Ad5/NZW rabbit ocular replication model. Ocular safety studies also determined whether CPE-C warrants further development as an antiviral agent for the treatment of adenoviral ocular infections.

## 2. Results

### 2.1. In Vitro Antiviral Testing (EC_50_ Determinations)

The in vitro antiviral testing was done using a standard plaque reduction assay with A549 human lung carcinoma cells as previously described [15,28]. The EC_50_s were determined using a Fitted Line Plot Regression analysis with Minitab Version 21 software. The EC_50_ is the effective concentration that inhibits plaque formation by 50%. The HAdV types tested represent the most common adenovirus serotypes that cause ocular infections (HAdV8, HAdV19/64, HAdV37 (EKC); HAdV3, HAdV4, HAdV7a (follicular conjunctivitis)) and serotypes that cause conjunctivitis and can replicate in the rabbit ocular model (HAdV1, HAdV2, HAdV5) [38]. The mean and standard deviation EC_50_ concentrations for CPE-C ranged from 0.030 ± 0.033 μg/mL (0.126 ± 0.138 µM) for HAdV5 to 0.059 ± 0.018 μg/mL (0.246 ± 0.076 µM) for HAdV8 (Table 1).

The CPE-C data compared favorably to those of a positive antiviral control, cidofovir (Table 1). The mean EC_50_ concentrations for cidofovir ranged from 0.427 ± 0.173 μg/mL (19.224 ± 0.491 µM) for HAdV8 to 5.367 ± 0.137 μg/mL (19.224 ± 0.491 µM) for HAdV7a with a majority of the HAdV serotypes demonstrating EC_50_ concentrations around 5 μg/mL. Overall, CPE-C demonstrated a narrower range of mean EC_50_ concentrations across the HAdV serotypes tested (2-fold) compared to the 12.6-fold differences demonstrated by cidofovir. CPE-C also demonstrated significantly lower EC_50_ concentrations of the cidofovir for each HAdV type except HAdV8 (Two-Sample T-Test). The EC_50_ concentrations were approximately 100-fold lower for CPE-C compared to cidofovir for a majority of the HAdV types (Table 1). The in vitro data suggests that the anti-adenoviral activity of CPE-C compares favorably to cidofovir, a proven potent ophthalmic topical antiviral in vivo [9].

### 2.2. In Vivo Ocular Toxicity in a Draize Rabbit Model

CPE-C was evaluated for ocular toxicity using the standard Draize ocular scoring method which has been in use to determine the toxicity of cosmetics and chemicals since 1944 [39]. CPE-C ointments (3%, 2%, 1%, 0.5%) were administered topically 4 times daily for 4 days in uninfected, non-scarified NZW rabbit eyes (*n* = 2). Additionally, the 3% CPE-C concentration was also evaluated in rabbit eyes after corneal scarification. Overall, CPE-C, in concentrations up to 3% in an Aquaphor vehicle, appeared to be non-toxic to NZW rabbit eyes. Specifically, CPE-C ointment in 3%, 2%, 1%, and 0.5% concentrations was non-irritating or practically non-irritating when administered to uninfected, non-scarified NZW rabbit eyes 4 times daily for 4 days (Table 2). 3% CPE-C ointment was practically non-irritating when administered to scarified NZW rabbit eyes 4 times daily for 4 days. There appeared to be no inhibition of corneal epithelial wound healing by the CPE-C in eyes that had undergone corneal epithelial scarification. Since the 3% CPE-C was the highest non-toxic concentration tested, this concentration was chosen for the in vivo antiviral efficacy testing in the Ad5/NZW rabbit ocular replication model.

### 2.3. In Vivo Antiviral Efficacy in Ad5/NZW Rabbit Ocular Replication Model

**Figure 2:**
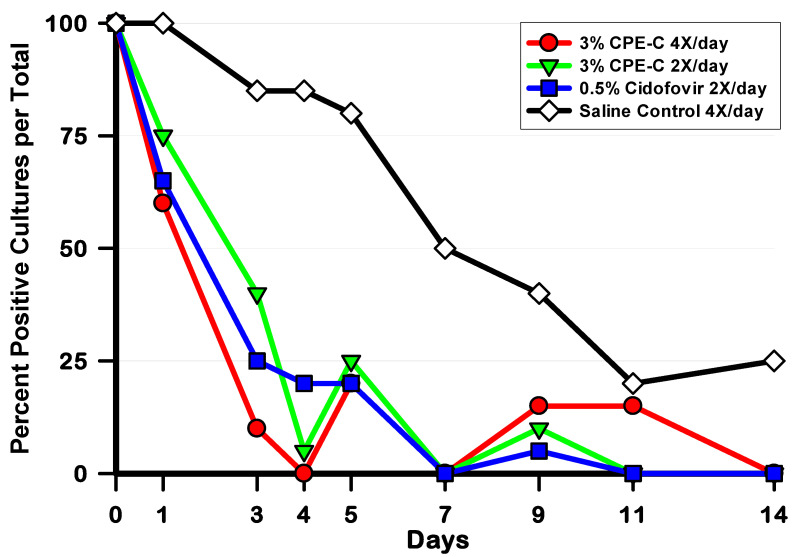
This figure presents the percentages of daily HAdV5-positive cultures per Total for each group. 3% CPE-C 4X/day, 3% CPE-C 2X/day, and 0.5% cidofovir 2X/day significantly reduced the number of HAdV5-positive cultures per group on days 1, 3, 4, 5, 7, and 14 (*p* ≤ 0.0471, FET) compare with the saline control. There were no significant differences among the antiviral treatments on any of the culture days. In addition, 0.5% cidofovir 2X/day had significantly fewer HAdV5-positive cultures on day 9 than the saline control (*p* = 0.0197, FET).

The results of the in vivo antiviral testing in the Ad5/NZW rabbit ocular replication model are summarized in Figure 2 and Table 3. Firstly, the daily number of HAdV5-positive culture per total was calculated for each group and culture day. The data were analyzed using the Fisher Exact Test (FET) and are displayed in Figure 2. All antiviral treatments (3% CPE-C 4X/day, 3% CPE-C 2X/day, and 0.5% cidofovir 2X/day) significantly reduced the number of HAdV5-Positive Cultures per Group on Days 1, 3, 4, 5, 7, and 14 (*p* < 0.05, FET) compare with the Saline Control. There were no significant differences among the antiviral treatment groups on any of the culture days. In addition, 0.5% cidofovir 2X/day produced significantly fewer HAdV5-Positive Cultures on Day 9 than the Saline Control (*p* = 0.0197, FET).

The HAdV5-positive cultures per total data were also analyzed using global measurements of HAdV5-positive cultures and these are presented in Table 3. The overall number of HAdV5-positive cultures per total for each antiviral treatment group throughout the experiment (days 1–14) were calculated and analyzed using the Chi-square test. 3% CPE-C 4X/day (24/160, 15%), 3% CPE-C 2X/day (31/160, 19%), and 0.5% cidofovir 2X/day (27/160, 17%) all significantly reduced the number of HAdV5-positive cultures per total (days 1–14) compared with the saline control (97/160, 61%) (*p* ≤ 0.011, Chi-square). We then divided the data into the early phase of infection (Days 1–5), during which the majority of viral replication takes place, and the late phase of infection (days 7–14) during which normal immune-mediated viral clearance takes place. During both the early and late phases of Infection, each antiviral treatment group demonstrated fewer HAdV5-positive cultures per total compared to the saline control (*p* ≤ 0.011, Chi-square). There were no statistically significant differences in the number of HAdV5-positive cultures per total among the three antiviral treatment groups overall and during the early and late phases of infection.

To analyze a global measure of HAdV5 replication during the early and late phases of Infection, the HAdV5 titers were combined for each group for each phase and analyzed for significant differences (Table 3). 3% CPE-C 4X/day, 3% CPE-C 2X/day, and 0.5% cidofovir all reduced the combined HAdV5 ocular titers compared to the saline control group in the early phase (*p* ≤ 0.004, ANOVA) and late phase (*p* ≤ 0.011, ANOVA) of infection. Again, there were no statistically significant differences in the antiviral activity among the three antiviral treatment groups (Table 3).

Finally, the mean duration of shedding (the last day on which a positive culture was obtained) is also presented in Table 3. The duration of shedding for each antiviral treatment group was all significantly shorter compared with the saline control (*p* ≤ 0.0001, ANOVA), and there were no statistically significant differences among the antiviral treatment groups.

## 3. Discussion

Pharmaceutical interest in developing an anti-adenoviral drug has increased greatly in recent years [9]. For commercial development as a topical ophthalmic anti-adenoviral, The Campbell Laboratory has argued that three criteria must be met by any candidate drug [15,19]: (1) demonstrated potent antiviral activity against a panel of HAdV serotypes that commonly infect the eye, (2) demonstrated significant antiviral activity in the Ad5/NZW rabbit ocular replication model, which serves as a surrogate for clinical infections [19], and (3) convincing topical and systemic safety data for future treatment of children and a prophylaxis indication.

The current study investigated the anti-adenoviral properties of CPE-C, a former anti-cancer drug candidate that was associated with patients’ deaths from refractory hypotension following intravenous administration [35]. Historically, CPE-C demonstrated impressive antiviral activity against a large, diverse group of RNA and DNA viruses, including HSV, CMV, VZV, yellow fever virus [24,32], and West Nile Virus [36]. As a CTP synthetase inhibitor, CPE-C belongs to an understudied class of antivirals that appears worthy of greater scientific attention [37]. The general mechanism of action is mediated through the inhibition of the enzyme CTP synthetase, the enzyme that converts UTP to CTP. This interaction results in a depletion of the CTP supply necessary for viral replication [32].

The potency of therapeutic agents is an important component of their pharmacokinetic and pharmacodynamic properties. In the case of topically applied ocular antiviral drugs, those with in vitro EC_50_ concentrations less than 10 μg/mL are considered to be potent antivirals. In addition, topical treatments to the ocular surface have the advantage of application of the drug directly to the affected tissue that can produce high therapeutic drug concentrations in ocular tissue which will improve the efficacy of the antiviral agent. Both of these characteristics are advantageous when evaluating a topical antiviral for ophthalmic use.

In the current study, CPE-C met the first criterion for development. It demonstrated potent antiviral activity against a panel of HAdV ocular types in vitro. CPE-C produced EC_50_ concentrations <0.06 µg/mL (<0.25 µM) across the panel of HAdV types that commonly infect the eye. The EC_50_s produced against a panel of adenovirus types that commonly infect the eyes were approximately 100-fold less for a majority of the HAdV types than those produced by the positive antiviral control, cidofovir, which has demonstrated antiviral efficacy in preclinical studies [5,6,7,8] and in phase 1 and 2 clinical trials in the USA for the treatment of adenoviral ocular infections [9]. These data provide a good indication that CPE-C will demonstrate antiviral activity in the Ad5/NZW rabbit ocular replication model.

In fact, that was the case. CPE-C met the second criterion for development as 3% CPE-C demonstrated potent antiviral activity in the Ad5/NZW rabbit ocular replication model. 3% CPE-C, administered 4X and 2X per day for 7 days, significantly decreased the number of HAdV5-positive cultures per total on days 1, 3, 4, 5, 7, and 14 compared with the Saline control. In addition, HAdV5 titers were significantly decreased during the Early and Late Phases of infection. Finally, the duration of shedding was significantly reduced by 6.8 days when 3% CPE-C was administered 4X per day for 7 days and by 5.6 days when 3% CPE-C was administered 2X per day for 7 days. The antiviral activity of CPE-C was comparable to that demonstrated by the antiviral positive control, 0.5% cidofovir. The reduction in viral titers during the acute infection produced by CPE-C should lead to decreased inflammation on the ocular surface in patients. This will lessen patient suffering and decrease the potential for the formation of vision-altering subepithelial corneal infiltrates. The significant decrease in the length of the infection would allow patients to return to school and work much more quickly than the recommended 2-week absence.

The third criterion, safety, was not completely evaluated in this study. Standard Draize testing of CPE-C in uninfected rabbit eyes demonstrated minimal toxicity in the ocular toxicity study. Topical treatment dose regimens used in vivo appeared to be acceptable from a safety point of view.

Like 2′,3′-dideoxycytidine (ddC; zalcitabine) [15], the most important unresolved issue affecting the future commercial development of CPE-C is systemic safety. The untimely deaths reported in 1995 of two cancer patients treated with intravenous CPE-C [35] raises serious concerns for the treatment of a self-limited non-blinding ocular infection. In the current rabbit studies, there were no obvious signs of systemic toxicity (decreased appetite, weight loss, wasting, death) following topical ocular administration of CPE-C in the doses given. However, these preliminary results do not definitively answer the questions of systemic safety following topical ocular administration of CPE-C. Therefore, the challenge for a pharmaceutical company will be to generate convincing and reassuring systemic safety data following topical administration. Given the negative clinical history associated with this compound, a persuasive package of unambiguous data will be required to convince appropriate governmental regulatory agencies to grant approval for the proposed ocular indication for CPE-C as the first topical anti-adenoviral drug.

## 4. Materials and Methods

### 4.1. Test Drugs

Cyclopentenylcytosine (MWT = 239.23) was provided by the Drug Synthesis and Chemistry Branch, Developmental Therapeutics Program, Division of Cancer Treatment and Diagnosis, National Cancer Institute. For the in vitro antiviral assays, CPE-C was dissolved in tissue culture media and diluted in media to the desired concentrations. For the in vivo studies, CPE-C (3%, 2%, 1%, 0.5%, *w*/*w*) ointments were prepared by the pharmacy at the University of Pittsburgh Medical Center by adding CPE-C to Aquaphor (a neutral, anhydrous base for compounding smooth, stable emulsions that contains 41% *w*/*w* petrolatum) in the appropriate concentrations. 25 drops of mineral oil were added to the emulsions in order to make them less stiff. 0.5% Cidofovir for the in vivo studies was prepared in IV saline from the 7.5% injectable form of cidofovir (Vistide, Gilead Sciences, Inc. Foster City, CA, USA). For the in vitro antiviral assays, the 7.5% injectable form of cidofovir (Cidofovir Injection, [Heritage Pharmaceuticals Inc., Eatontown, NJ, USA]) was diluted in tissue culture medium to the desired concentrations. Saline for intravenous use (Baxter, Deerfield, IL, USA) served as control drops for the in vivo studies.

### 4.2. Adenovirus Isolates and Cells

For the in vitro antiviral testing, clinical adenovirus isolates of types HAdV1, HAdV2, HAdV3, HAdV4, HAdV5, HAdV7a, HAdV8, and HAdV19/64 were anonymously collected from patients presenting with typical adenoviral conjunctivitis at the Charles T. Campbell Ophthalmic Microbiology Laboratory at the UPMC Eye Center, University of Pittsburgh, Pittsburgh, PA, USA. The isolates were retrieved from a frozen −80 °C retrospective clinical collection that was de-identified and stored for diagnostic test validations. The types of isolates were determined using serum neutralization [30]. Recently, HAdV19 has been reclassified as HAdV64 [41]; however, for the purpose of this study, it was designated as HAdV19/64. No clinical isolates of HAdV37 were identified, therefore the ATCC (American Type Culture Collection, Manassas, VA, USA) reference strain of HAdV37 was used. The clinical isolates along with the ATCC HAdV37 reference strain were grown in A549 cell (CCL-185, ATCC) monolayers and stocks were prepared, aliquoted, and frozen at –80 °C. The titers of the stock viruses were determined as described in Section 4.7. The same clinical isolate of HAdV5 used for the in vitro antiviral testing was also used for the in vivo antiviral evaluation in the Ad5/NZW rabbit ocular replication model [5,6,7,8,15,16,19,28,29,30].

A549 human lung carcinoma cells were grown and maintained in Eagle’s MEM supplemented with 10% fetal bovine serum (Sigma Cell Culture Reagents, St. Louis, MO, USA). The A549 cell line was used to prepare the adenovirus stocks and for the determination of viral titers in the Ad5/NZW rabbit ocular replication model and in the in vitro plaque reduction assays.

### 4.3. Animals

Two-to-three-pound female New Zealand white (NZW) rabbits were purchased from Myrtle’s Rabbitry, Thompson Station, TN, USA. All animal studies conformed to the ARVO Statement for the Use of Animals in Ophthalmic and Vision Research. Approval was obtained from the University of Pittsburgh Institutional Animal Care and Use Committee (IACUC) prior to the initiation of the studies (Protocol #0502953).

### 4.4. In Vitro Antiviral Testing (EC_50_ Determinations)

Plaque reduction assays were performed 2–4 times using 24-well multiplates containing A549 monolayers. One plate per virus isolate per drug was used. The 24-well multiplates were inoculated with approximately 100 plaque-forming units (PFU) of HAdV per well. After a three-hour adsorption period, the inocula were removed from all wells. One ml of overlay media with 0.5% methylcellulose containing 100, 10, 1.0, 0.1, 0.01, or 0.001 μg/mL of CPE-C or cidofovir was added to 3 wells each. To the remaining 6 wells, 1 mL of overlay media with 0.5% methylcellulose and without CPE-C or cidofovir was added. The plates were incubated at 37 °C in 5% CO_2_ until plaque formation was visible. At that time, the cells were fixed and stained with 0.5% gentian violet containing formalin. The plates were dried, and the number of plaques per well was counted using a dissecting microscope. The CPE-C and cidofovir EC_50_ concentrations (concentrations that inhibits plaque formation by 50%) for each virus isolate were determined using a Fitted Line Plot Regression analysis (Minitab Version 21, State College, PA) of the average number of plaques versus drug concentration. The mean and standard deviation EC_50_ concentrations of 2–4 trials were then determined for each HAdV isolate. The EC_50_ concentrations in μg/mL were then converted to μM using the GraphPad online Molarity Calculator (https://www.graphpad.com/quickcalcs/Molarityform.cfm (accessed on 12 June 2023)).

In general, for topical ophthalmic applications that can produce high tissue concentrations in ocular tissue, antiviral agents with EC_50_ concentrations less than 10 μg/mL are considered to be potent antivirals. Antivirals demonstrating EC_50_ concentrations between 10 and 50 μg/mL are considered to be moderately potent while agents that have EC_50_ concentrations between 50 and 100 μg/mL are considered minimally potent. EC_50_ concentrations of greater than 100 μg/mL signify little or no antiviral activity.

### 4.5. Ocular Toxicity in Draize Rabbit Model

CPE-C ointment (3%, 2%, 1%, 0.5%) was administered topically 4 times daily for 4 days in uninfected, non-scarified NZW rabbit eyes (*n* = 2). The CPE-C ointments were administered in approximately ½ inch strips per dose via a syringe with a 19-gauge cannula attached. The eyes were closed and gently rubbed to produce even distribution of antiviral over the entire ocular surface. The 3% CPE-C concentration was also evaluated in rabbit eyes after corneal scarification (general anesthesia with 40 mg/kg of ketamine and 4 mg/kg of xylazine administered intramuscularly and topical ocular anesthesia with proparacaine followed by 12 cross-hatched strokes with a #25 needle). All eyes were evaluated in a masked fashion for ocular toxicity using the Draize and MMTS scoring system [37,38] on days 2, 3, and 4.

### 4.6. Ad5/NZW Rabbit Ocular Replication Model for In Vivo Antiviral Efficacy Testing

Using the Ad5/NZW rabbit ocular replication model [5,6,7,8,15,16,19,28,29,30], the current study was performed in duplicate trials using a total of 40 rabbits (20 per trial). Following the systemic and topical anesthesia described above, the rabbits were topically inoculated with 50 µL (1.5 × 10^6^ PFU/eye) of HAdV5 in both eyes after corneal epithelial scarification (12 cross-hatched strokes of a #25 sterile needle). Twenty-four hours later, the rabbits were randomly assigned to one of four topical treatment groups (*n* = 10): (1) 3% CPE-C 4X/day, (2) 3% CPE-C 2X/day, (3) 0.5% Cidofovir 2X/day, and (4) Saline Control 4X/day. Rabbits were treated topically in both eyes according to the above treatment regimens for 7 days. The CPE-C ointments were administered as described above in the ocular toxicity study. Cidofovir and saline were administered as 37 µL aqueous drops. Following topical anesthesia with 0.5% proparacaine, ocular swabbing to recover HAdV5 from the tear film and corneal and conjunctival surfaces was performed on days 0, 1, 3, 4, 5, 7, 9, 11, and 14 after inoculation. The cultures were taken at least 1 h after the final dose of antiviral on the days the antivirals were administered. The swabs from each eye were placed individually into tubes containing 1 mL of tissue culture media and were frozen at −80 °C pending viral plaque assay.

### 4.7. Determination of Viral Titers Using the Viral Plaque Assay

The samples to be assayed were diluted 1:10 for up to 6 dilutions. One-tenth of a ml of both the undiluted sample and the dilutions was inoculated onto duplicate wells of 24-well multiplates containing A549 monolayers. The virus was adsorbed for 3 h at 37 °C in a 5% CO_2_-water vapor atmosphere. The plates were rocked intermittently to keep the cells from drying. Following adsorption, 1 mL of tissue culture media containing 0.5% methylcellulose was added to each well, and the plates were incubated at 37 °C in a 5% CO_2_-water vapor atmosphere. After the appropriate incubation period, the cells were fixed and stained with 0.5% gentian violet in formalin. The plates were dried, and the number of plaques per well was counted under a dissecting microscope. The viral titers were then calculated and expressed as plaque-forming units per milliliter (PFU/mL).

### 4.8. Statistical Analysis of In Vivo Efficacy Studies

The in vitro EC_50_ data was analyzed with Two-Sample T-Tests using Minitab (Minitab Inc., State College, PA, USA) statistical software Version 21. Following the completion of both trials in the in vivo antiviral efficacy study, the data from each trial were analyzed statistically using Minitab and GraphPad online (https://www.graphpad.com/quickcalcs/contingency1/ (accessed on 16 February 2023)). As comparable results were obtained in each trial, the data were then pooled to obtain a larger subject number and analyzed using Analysis of Variance (ANOVA) with Fisher’s pairwise comparisons (Minitab), Chi-Square (Minitab), and Fisher Exact Test (GraphPad online). Significance was established at the *p* ≤ 0.05 confidence level.

## 5. Conclusions

From the results of this study, we conclude that the CTP synthetase inhibitor, cyclopentenylcytosine (CPE-C), demonstrated potent in vitro antiviral activity against a panel of ocular adenovirus types that commonly infect the eye. In vivo, topically applied 3% CPE-C was non-toxic to naïve and scarified rabbit eyes and was an effective inhibitor of HAdV5 replication in the Ad5/NZW rabbit ocular replication model.

## Figures and Tables

**Table 1:** In vitro CPE-C and cidofovir antiviral testing using a plaque reduction assay.

Mean and Standard Deviation EC_50_ Concentrations of CPE-C and Cidofovir					
	CPE-C		Cidofovir		
HAdV Type	EC_50_ [μg/mL]	EC_50_ [μM]	EC_50_ [μg/mL]	EC_50_ [μM]	*p* *
HAdV1	0.051 ± 0.007	0.214 ± 0.027	5.268 ± 0.588	18.87 ± 2.110	0.004
HAdV2	0.047 ± 0.024	0.198 ± 0.101	5.106 ± 0.410	18.29 ± 1.469	0.002
HAdV3	0.040 ± 0.004	0.165 ± 0.016	4.119 ± 0.910	14.75 ± 3.260	0.016
HAdV4	0.032 ± 0.026	0.134 ± 0.110	4.605 ± 0.925	16.49 ± 3.310	0.013
HAdV5	0.030 ± 0.033	0.126 ± 0.138	4.921 ± 0.486	17.63 ± 1.740	0.045
HAdV7a	0.055 ± 0.026	0.194 ± 0.057	5.367 ± 0.137	19.22 ± 0.491	0.012
HAdV8	0.059 ± 0.018	0.246 ± 0.076	0.427 ± 0.173	1.556 ± 0.592	0.060
HAdV19/64	0.047 ± 0.028	0.196 ± 0.118	4.898 ± 0.517	17.54 ± 1.850	0.004
HAdV37	0.048 ± 0.008	0.202 ± 0.037	4.790 ± 0.678	17.16 ± 2.430	0.007

* *p*-value of Two-Sample T-Test comparing the EC_50_ concentrations of CPE-C and cidofovir in μg/mL for each HAdV type.

**Table 2:** In vivo ocular toxicity in Draize rabbit model [39].

Maximum Mean Total Score [40]			
Group	Day 2	Day 3	Day 4
3% CPE-C	0.0—N	2.0—PN	2.0—PN
2% CPE-C	0.0—N	0.0—N	0.0—N
1% CPE-C	0.0—N	0.0—N	0.0—N
0.5% CPE-C	1.0—PN	1.0—PN	1.0—PN
3% CPE-C *	1.0—PN	1.0—PN	1.0—PN

* = Eyes with Scarification; N = Non-Irritating; PN = Practically Non-Irritating.

**Table 3:** In vivo antiviral efficacy in Ad5/NZW rabbit replication model.

	Saline Control	3% CPE-C	3% CPE-C	0.5% Cidofovir
	4X/Day	4X/Day	2X/Day	2X/Day
HAdV5-Positive Eye Cultures/Total				
Overall (Days 1–14)	97/160 (61%)	24/160 (15%) ^a^	31/160 (19%) ^a^	27/160 (17%) ^a^
Early Phase (Days 1–5)	70/80 (88%)	18/23 (21%) ^a^	29/80 (36%) ^a^	26/80 (33%) ^a^
Late Phase (Days 7–14)	27/80 (34%)	6/80 (8%) ^a^	2/80 (3%) ^a^	1/80 (1%) ^a^
Mean ± Sd Combined HAdV5 Ocular Titers (Log_10_ PFU/mL)				
Early Phase (Days 1–5) (*n* = 80)	1.0 ± 3.0 × 10^2^	1.6 ± 4.8 × 10^1 b^	3.5 ± 18.1 × 10^1 b^	6.7 ± 12.7 × 10^0 b^
Late Phase (Days 7–14) (*n* = 80)	2.2 ± 9.2 × 10^1^	0.9 ± 4.4 × 10^0 c^	3.6 ± 25.2 × 10^0 c^	0.3 ± 2.2 × 10^0 c^
Duration of HAdV5 Shedding (Days)				
Mean ± Sd (*n* = 20)	8.1 ± 3.4	1.3 ± 1.6 ^d^	2.5 ± 2.3 ^d^	2.0 ± 1.8 ^d^

^a^ *p* ≤ 0.011 compared to Saline Control (Chi-square) ^b^ *p* ≤ 0.004 compared to Saline Control (ANOVA) ^c^ *p* ≤ 0.011 compared to Saline Control (ANOVA) ^d^ *p* ≤ 0.0001 compared to Saline Control (ANOVA).

## Data Availability

The data reported in this study are available in this manuscript.

## Sample Availability

Samples of the compounds are not available from the authors.
